# Covid-19 as a Social Crisis and Justice Challenge for Cities

**DOI:** 10.3389/fsoc.2020.583638

**Published:** 2020-11-12

**Authors:** Annegret Haase

**Affiliations:** Department of Urban and Environmental Sociology, Helmholtz Centre for Environmental Research – UFZ, Leipzig, Germany

**Keywords:** Covid-19, cities, social crisis, justice challenge, urban sustainability

## Abstract

The article deals with Covid-19 as a social crisis and justice challenge for cities. It describes how Covid-19 shines a spotlight on the uneven distribution of goods and burdens, opportunities and resources that we find in most of the world's cities today; inequality and justice challenges arise from both the crisis itself and some of the policy reactions to it, such as the stay-at-home orders and economic lockdowns. It shows how exposure and vulnerability to Covid-19 emerges mainly at the intersection between different dimensions of disadvantage and marginalization. The example of housing and green space provision is used to discuss this general argument in more detail. The article concludes that to overcome the social crisis and justice challenge posed by Covid-19, we have to tackle the underlying structures/mechanisms leading to inequitable outcomes in today's cities, and to re-think the social and justice yardsticks of current urban sustainability and resilience debates and strategies.

## Introduction: The COVID-19 Crisis as an Urban Social Crisis and Justice Challenge

Cities^1^ worldwide have become the hotspots of the current Covid-19 crisis. The pandemic represents, in a way, an urban crisis and challenge for thinking about and shaping sustainable urban futures. It has affected cities in a very unequal manner: There are huge differences between different countries and between cities within one country, in regions all across the globe. But there are also some common features that become obvious when we look at the “urban face” of the Covid-19 crisis. The degree to which the pandemic affects a place and exposes people to health risks is clearly related to the social and socio-spatial inequalities and inequities of the world's cities. As Simon (2020) rightly states: “Patterns of illness and death reflect urban social and economic geographies. Attention has focused on shielding the elderly and those with underlying medical conditions, defined as being most at risk, but the reality is more complex.” Clearly, and this is nothing really new, low socio-economic status is another huge risk factor, a fact that holds true for all regions of the world (Fisher and Bubola, 2020). Both the health crisis itself and the measures taken to counteract it (e.g., restricting people from leaving their homes and shutting down large parts of public and economic life including supply and care infrastructures) not only clearly show existing social, socio-economic and socio-spatial inequities in our cities. They also reinforce and strengthen those inequities, leading to higher exposure to the virus among the disadvantaged and leaving them less capable of protecting themselves from infection. It became clear that social risk factors such as poverty, precariousness and exclusion increased a person's likelihood of becoming infected with the virus. Friedmann and Bartsch (2020) even speak of SARS-CoV-2 as a “social virus.” They identified poverty and exclusion as global risk factors that influence whether urban dwellers become infected, suffer from a severe case of the disease and even die from it. In countries like the US, Brazil, the UK or India, just to mention a few examples, the share of poor and low-income people among the Covid-19 cases and fatalities is much higher than their general share in the population. Hence the more socially polarized cities are, the more probable is it that this polarization will be reflected in the figures regarding who is exposed to and affected by the virus (Fisher and Bubola, 2020; Friedmann and Bartsch, 2020). Obviously, the social crisis is most severe in those (urban) societies with the most uneven distribution of and access to opportunities, resources and safety for different population groups and, as a consequence, the greatest social polarization—as witnessed in the countries mentioned above.

Set against this background, in this short article I will look at the coronavirus pandemic as a social crisis and justice challenge for our cities and urban societies. I will first explain what I mean by “social crisis” and “justice challenge” and then specifically discuss how disadvantage affects (a) exposure to the virus and (b) vulnerability to suffer directly and indirectly from the crisis. I will take the empirical example of housing and green space provision and distribution in cities to explore this in more detail. Finally, I will discuss how we can proceed from here to transform our cities in a truly sustainable, inclusive and just way to make them more resilient against external shocks and challenges.

Why does Covid-19 represent a social crisis in our cities? It is a social crisis in three senses, since it (a) exposes and highlights existing social inequities and shortcomings; (b) reinforces or enlarges them, because policies designed to counteract the crisis create unequal (polarized) opportunities for people to adapt and protect themselves; (c) has great potential to continue amplifying social differences even after the lockdowns end, i.e., also leads to more inequalities and inequities in the long run. The Covid-19 crisis is also a “stressor for justice issues,” as Kotsila (2020) recently put it. The opportunities and constraints that influence how well people get through the pandemic—economically and with regard to health—are distributed in a very unjust manner. It is not only the case that the poorest and most excluded are hardest hit by the pandemic; in some respects, the response to the crisis also comes at the expense of those with the lowest incomes, the worst housing conditions, and the least access to high-quality open/green space, health insurance, healthy food, etc. The latest data shows that social polarization did not just become obvious in relation to exposure to the virus, vulnerability to infection and the capacities of people to adapt to lockdown conditions; in the longer term, forecasts suggest we can expect a further increase in disadvantages for those who are most vulnerable and hardest hit by the crisis in a social respect. This polarization is concentrated in cities (DGB, 2020; Fisher and Bubola, 2020). In the following, I will discuss how the Covid-19 pandemic and policy responses to it have reinforced the social crises that already existed in cities, and show how crucial it is to apply an intersectional perspective to grasp the complexity of this crisis. The way urban societies have responded to the health crisis up until now also shows how inequitable our policy responses are for urban dwellers who are marginalized or suffer low incomes or bad housing and living conditions, and how both the pandemic and the political response to it run the risk of considerably aggravating the social crises in our cities.

Conceptually, this health crisis and how it has unfolded until now (autumn 2020) is the focus of multiple, overlapping debates with great potential for cross-fertilization. The social crisis, as I understand it here, touches different strands of the debates at their intersections:

- It represents a real challenge for large-scale debates on the future of urban sustainability and urban transformation toward greater sustainability. Having said that, I see this issue as another contribution to the current critical discussion on the “ambition-reality gap” of urban sustainability and the rising demands to pay more attention to the social and justice dimensions of the general sustainability discourse (Agyeman et al., 2002; Agyeman, 2008; Kremer et al., 2020).
- It points to the urgent need to deal more critically with social and justice challenges in debates about urban transformation and the ways in which greener, more affordable and healthier cities for all can be designed and implemented. In the last few years, there has been an increase in critical analysis dealing with the awareness of inclusivity and justice in environmental urban debates (Haase et al., 2017; Malin and Ryder, 2018; Menton et al., 2020). More and more, we find overlaps between such critical studies and the ongoing right-to-the-city debates that started decades ago with the work of Lefebvre, Harvey and Marcuse, and which did not end with Sassen's work on the city as a “collective good” 2017 or Fainstein's “Just City” 2010.
- It tells us a lot about how our societies cope with social and justice challenges in times of crisis, and whether we can interpret the crisis itself as a condition that is aggravating gaps in equality and justice or as a chance to tackle and overcome existing problems. In a situation of risk and uncertainty that puts society under stress—as conceptualized by Beck (1986) and recently recalled by Nassehi, among others (Nassehi and Yüksel, 2020)—the way this “crisis condition” shapes and determines how we handle inequality and justice issues says a lot about the way a society treats social issues (Are social issues a central priority, dwarfed by other issues or ignored altogether?).

In light of this, it is not just important to analyse the social and justice dimensions of how the Covid-19 crisis emerged in and is affecting our cities. It is equally or even more important to discuss what this means for our conceptual claims on the futures of urban sustainability and the formulation of policies and their implementation. So, my argument includes both an empirical foundation and observations as well as a theoretical and policy-related reflection. This paper is not an empirical one, it delivers a perspective based on my empirical expertise in urban research so far, observations I've made during the last few months, a study of the emerging discourse on the urban dimension of the coronavirus crisis, and exchanges within my scholarly networks. The section on housing and the provision of green space builds on my nearly two decades of expertise and experience in this field. My aim is to open up a perspective on Covid-19 as a social crisis and justice challenge for cities and generate discussion.

## Vulnerability and Exposure at the Intersection of Disadvantages

“Poor people also inhabit the lowest quality housing and areas of a city. They live at the highest densities and in the most cramped accommodation. These areas have higher air pollution levels, and poor quality or inaccessible utilities and services. They often have the smallest areas of open public spaces.” (Simon, 2020) This quote is part of a small but increasing number of studies and reflections on the impacts of the Covid-19 pandemic (on cities). It shows the heart of what I call the social crisis and justice challenge caused by Covid-19. The social dimension of risk and exposure was not in the center of attention when the crisis started. It took researchers a while to grasp it. While at first, risk and exposure were mainly discussed with respect to age and pre-existing illnesses, it quickly became apparent that there are also social risk factors such as poverty, bad housing and living conditions, crowded areas and neighborhoods, job precariousness, lack of access to health services, and the necessity of going to work during lockdowns. Many of those studies and reflections (e.g., Friedmann and Bartsch, 2020; Schneidewind et al., 2020; Simon, 2020; Teubner, 2020) share the opinion that the crisis brings to light a range of (existing) inequalities. As Simon (2020) rightly puts it: “…the combination of social, economic and demographic factors together with the urban environment probably accounts for many of the observed infection patterns.”

I think an intersectional perspective is needed here to fully grasp the complexity of exposure and disadvantage (Crenshaw, 2000; Lutz, 2014). Multiple disadvantages also mean that especially vulnerable people

- experience above-average exposure to external risks, either caused directly by the Covid-19 crisis or the policy response to it;
- either lack or do not have equal capacities to adapt or protect themselves (the way shutdowns or restrictions are organized or implemented limit their capacities even more, e.g., stay-at-home orders, rules about leaving one's home or district, closure of social support infrastructure);
- are also most likely to suffer from the long-term consequences of the crisis through the loss of jobs, decreased income, sustained and untreated health problems, as well as longer educational and professional career gaps.

So, it is not just the intersection of disadvantages itself that makes people specifically vulnerable, but also the temporal dimension that keeps them disadvantaged in the long run. It is what Fisher and Bubola (2020) call a “mutually reinforcing cycle” of existing inequalities and external threat that make Covid-19 a social crisis for cities. Even worse, “inequality itself may be acting as a multiplier on the coronavirus's spread and deadliness. Research on influenza has found that in an epidemic, poverty and inequality can exacerbate rates of transmission and mortality for everyone.” (Fisher and Bubola, 2020). The widening of inequalities may additionally widen societal gaps that drive polarization, radicalism and right-wing populism in the long run as we see it currently in the US and Brazil.

Multiple disadvantages look different across the globe, we find a lot of differences and factors that are specific to particular places, but we also find some common ground which I described above. Intersectional disadvantage as the “face” of the urban social crisis in times of Covid-19 is a global phenomenon. It affects seasonal workers in Europe and India, precarious workers who lost their jobs in the US, people living in crowded and poor areas without access to clean water, space for distancing or access to open/green space in the midst or at the fringes of our cities, refugees and people without a secure legal status, homeless people, etc. (e.g., Dick, 2020; Global Platform for the Right to the City, 2020). In order to describe a bit more in detail what I have outlined now in a general way, I will now expand on the field of housing and green space provision in cities.

## Examining the Example of Housing and Green Space Provision

The field of housing and green space provision shows the possible outcomes of crises in cities where there is an uneven distribution of and access to resources, opportunities and safety. Housing and green space provision belong to the fundamental issues that shape and determine people's quality of life and safety in cities. The case of the Covid-19 crisis has so far shown (a) how differently people were hit by the risk of infection depending on their housing conditions and their access to green spaces and (b) that the policies to counteract Covid-19, especially the conditions of lockdown, have by and large aggravated those uneven conditions. Socio-spatial and socio-economic inequalities were mirrored in the crisis and enlarged by the policy response—so the more polarized a city's living conditions, the greater the extent of the inequities.

Examples from many cities show that exposure to the risk of becoming infected is higher for residents living in crowded circumstances, in dense housing, small flats without a balcony and small rooms/little space per person. In our cities, it is mainly income-poor people who live under such conditions. As Simon (2020) underlines for the global scale, crowded housing has become a risk factor. What is more, in many places low-income housing is also situated in urban areas with high levels of pollution, e.g., along main traffic axes. Poor people often have an above-average level of pre-existing illnesses, which makes them additionally vulnerable. The lockdowns did not change this: They simply transferred the risk of getting infected from public spaces to households and indoor areas. Chair (2020) rightly speaks of the “inequalities of stay home policy,” because for people living in bad housing conditions staying at home means being “exposed to cold, damp and other hazardous conditions with consequences for both physical and mental health,” often with too little space per person (also: Clifford, 2020) and poor or no opportunities to access high-quality open and green spaces. Working from home or home schooling were hardly possible under such conditions, which risked further widening the existing gap in terms of opportunities for escaping poverty and marginalization. Precarious mass housing such as refugee accommodation or overbooked flats for seasonal workers became hotspots of mass infections in many cities, as examples from many countries show (e.g., Heisterkamp and Sussebach, Sauerbrey, 2020 for Germany, Butler, 2020, Chair, Clifford, 2020 for UK, Wennberg, 2000 for Sweden, Williams, 2020 for the US). The decisions of local governments in Spain to close down poor neighborhoods that had become infection hubs (e.g., in Palma de Mallorca or Madrid) in early autumn 2020 deepened the disadvantage of people living there (e.g., Jones, 2020). Not to mention homeless people for whom staying at home is not an option at all. All those groups of poor and/or marginalized people have suffered additionally from the closure of social support infrastructure, particularly children, single mothers and refugees/migrants with a fragile legal status. Care and help became hard or even impossible to provide during the lockdowns. With respect to jobs, many income-poor people had to either continue working and were exposed to the risk of infection in times when others could more effectively protect themselves by working from home. For many people, the economic lockdown meant a considerable loss or reduction in income that led to difficulties in paying rent, among other things. The moratoria on rent payments that was established in many countries only postpone the problem of indebtedness; evictions and the threat of eviction will remain a long-term disadvantage for poor people (e.g., Maalsen et al., 2020). In countries without a (functioning) welfare system, the crisis has made food more expensive due to lockdown conditions or disturbed supply chains. This, too, affects low-income people first and foremost. The interdependency or relatedness/interaction between all of these factors has to be understood to really grasp the extent to which the crisis and the associated political response have led to greater marginalization of already marginalized people in cities.

This is also true for the use of or access to urban green spaces. It is not only that urban green spaces are unevenly distributed in most cities and better-off housing areas and households usually benefit more from high-quality green spaces than poorer households and less popular housing areas. The urban green space debate rightly states that “during these extraordinary circumstances, urban nature offers resilience for maintaining well-being in urban populations, while enabling social distancing” (Samuelsson et al., 2020). Yet this remains an “empty” statement when we think about the uneven distribution of green benefits in most of our cities; this thinking would benefit from the insights of research that explicitly deals with green justice challenges (Haase et al., 2017; Kronenberg et al., 2020; Langemeyer and Connolly, 2020) or with the challenge of prioritizing the needs of the most marginalized (Anguelovski et al., 2020). To be very clear: The structures and organization of access to and benefits from urban green spaces that do not provide equitable outcomes in non-crisis conditions (as described in many studies by critical urban green scholars) will be even less equitable in crisis conditions. In recent weeks and months many surveys have been conducted that ask urban dwellers worldwide about changes in the way they use urban green space. These surveys will most probably not be able to show the inequities and mismatches between the needs and wants from the perspective of the under-privileged, since they will have hardly participated in such surveys. So, again, it is important to stress the crucial function of urban green spaces as resilient infrastructure that provides safe and quality places for people in times of pandemics and pandemic-related lockdowns—however this is not the full picture. It is also vital to consider the varying degrees to which different groups can make use of and enjoy (discrimination-free) access to such green spaces. A combined “green and justice” perspective would tackle the issue of green space access and benefits as a justice challenge in a more appropriate way; critical urban green discourse increasingly points to such engaged positions (Haase et al., 2017; Anguelovski et al., 2020; Kronenberg et al., 2020; Langemeyer and Connolly, 2020; TNOC Blog Roundtable on Covid-19, 2020). Here, there needs to be more focus on the importance of accessible allotment gardens at affordable prices, the improvement of small or unused green spaces in crowded neighborhoods (green courtyards, pocket parks, corner green, etc.) or the potential to create green roofs as spaces to spend time in low-cost housing areas, among others (e.g., Samuelsson et al., 2020). Last but not least, this also applies to spaces for urban farming as places of low-budget food production that also became more important during the crisis.

Large-scale, fundamental efforts will be required to improve housing and green spaces provision in cities in a way that creates more equitable results in general, but also in times of crisis. Those efforts would also include tackling housing market mechanisms, property rights and income issues. Such efforts would have to be based on the basic principles of a right of all urban citizens for safe and good housing and discrimination-free access to urban green spaces at the neighborhood level. The current situation in most cities shows how far away we are from such a reality, even from such thinking. The reaction to the crisis can veer toward many different directions. It may either enlarge existing inequality and justice gaps or improve the situation for the disadvantaged as a “lesson learnt” from the crisis for more resilience in the future. In some places, as mentioned, highly affected poor neighborhoods are simply closed to fight the spread of disease and infections. In other places, planned or existing greening projects received more support and were fast-tracked in cities, and there was critical debate about precarious housing (e.g., debate in Germany about housing for seasonal or contract workers) (Scheiwe, 2000). But these issues are tackling the defective appearances of an inequitable system, fighting against the worst symptoms, rather than tackling the problems at heart of the system: the unequal and unjust distribution of resources, opportunities, safety and quality of life in our cities. Or perhaps this assessment is too pessimistic? Maybe the Covid-19 crisis and the small improvements and changes in practices and discourse are the start of larger-scale and more fundamental changes toward truly sustainable cities (Kotsila, 2020; Kremer et al., 2020)?

## Discussion: How Do We Go Further From Here to Tackle the Social Crisis and Justice Challenge in Our Cities (Re)produced by COVID-19?

It remains unclear what this all means for

- the discourse on sustainable urban future cities in terms of a fair distribution of and access to opportunities, resources, quality of life and safety for all;
- future discourse including different strands of debate with a particular focus on their overlaps and cross-fertilization potential;
- policy formulation, planning and implementation that aims to tackle, counteract and, in the long run, overcome the social crises that we are observing in cities today as a consequence of the Covid-19 crisis.

There are various directions in which cities could develop during and after the pandemic. Under the given circumstances, it is not very likely that the inequalities and inequities that led to the socially uneven distribution of vulnerability and exposure to the virus will be tackled on a larger scale. This is where engaged, sustainability-oriented social science has to jump in and demonstrate how inequalities might emerge as a long-term risk for urban societies in the long run. For instance, there are a lot of green urban scholars who demand a more critically and emancipatory approach to discussions on sustainable and inclusive urban futures (e.g., Anguelovski et al., 2020), or plead for environmental science to take more responsibility for social concerns. So, if we want to see a different future “after” Covid-19 and not just a step-by-step return to the neoliberal and unjust “normality” that existed until early 2020, we have to demand fundamental changes to the shape and organization of our cities, and beyond. To this end, it would be desirable and indeed vital to deal more stringently and rigorously with overlaps between the strands of discussion on sustainable urban transformation and the right to the city, and raise more awareness about inequalities and justice among green urban scholars as I mentioned at the beginning. The policy fields that I briefly expanded on—housing and green space provision—are good examples for the potential of cross-fertilization between critical, socially aware and justice-oriented perspectives of urban discourse and research.

Clearly, the Covid-19 crisis offers a window of opportunities for change toward more sustainability. It might trigger or speed up changes that otherwise wouldn't have come so quickly, or make room for suggestions and decisions that promote a more sustainable way of looking at globalization, global urbanization, biodiversity protection and climate change mitigation, etc. In some places, efforts to strengthen green infrastructure and space for people increased during and after the lockdown periods, and some relief was provided for people who could not pay their rent due to job losses, reduced working hours and loss of income. The general question, however, is who will benefit and suffer from changes and opportunities resulting from the Covid-19 crisis, and whether the crisis will lead to opportunities for different groups of people in the long run. A crucial lesson that we have learnt from the pandemic is that we need to ensure that policy responses to crises (and the subsequent recovery periods) are also equitable and affordable for people with fewer opportunities, resources and capacities to react/adapt (see here also Teubner, 2020). Otherwise, future cities will neither be truly sustainable nor resilient in a socially responsible way. So, after all, it would be somewhat naïve to deny that to take the Covid-19 crisis as an opportunity depends on a variety of conditions, conditionalities and contingency.

If we go one step further here, we arrive at a point where we have to seriously look at how sustainability policies are being lived and implemented in our cities today. We see a large gap between our ambitions and the reality, a discrepancy that is highlighted when we examine the increased social crises caused by Covid-19. To tackle this gap, we have to fundamentally question how we measure sustainable (and resilient) cities. What role should social fairness and justice play here? Are we willing to learn from the way the social crisis reveals increased virus exposure and vulnerability at the intersection of disadvantages? Can we develop a policy that tackles aggregated marginalization? Which role can and should an engaged and critical global urban discourse play here? Recent debates call on the “planetary” scientific community to take an active role (Mukherjee, 2020) and also demand close communication between scientific research and practice, as has been the case with virology and epidemiology in the last few months.

Counteracting the social crisis and taking up the justice challenge (re)produced by the coronavirus in our cities represents a multi-level and multi-temporal endeavor. The crisis exists at all levels, from single households to whole countries. The city itself is a place where different levels of impact and affectedness come together and interact. The same is true for the short-term and long-term effects and changes. Hence the social crisis has to be considered and tackled with regard to the short-term and the longer-term. As Samuelsson et al. (2020) point out in relation to urban green space planning, short-term action would be needed to build/improve resilience during the crisis, and long-term action to re-arrange structures and organizational approaches in a way that provides more equitable outcomes and less polarized access to resources, opportunities and safety, regardless of whether there is a crisis. To put it another way: While it will not be possible to overcome all inequitable structures and modes of distribution in the short term, over the longer term it should be possible to minimize the risks of exposure and vulnerability for everyone, especially disadvantaged and marginalized groups (this represents a long-standing key argument in philosophical and social justice thinking since the time of John Rawls).

In his plea for urban social inequities to be made the focus of debate, Simon (2020) concludes: “We have a unique opportunity to work toward fairer, more sustainable cities in the wake of coronavirus. Emergency government […] support packages must be used proactively.” I fully share this view and would add by way of conclusion that we also need to take Covid-19 as proof of where and how the social reality in our cities is not sustainable, just or resilient. Inequalities and inequities are major obstacles to true sustainability and not just collateral damage or by-products that have to be accepted. Thus, we can also use the coronavirus crisis as an opportunity to re-think and re-discuss the type of sustainability we want to see in our cities in the future as well as the criteria for measuring it. And hopefully this re-think will have a string focus on counteracting and overcoming the social crisis and justice challenge currently being highlighted and reproduced by Covid-19.

With this short piece I sought to shape some ideas or at least thoughts and questions about these fundamental questions and invite further debate.

## Author Contributions

The author confirms being the sole contributor of this work and has approved it for publication.

## Conflict of Interest

The author declares that the research was conducted in the absence of any commercial or financial relationships that could be construed as a potential conflict of interest.

## Footnotes

^1^When referring to cities, I mean the city as an administrative area, but also its functional sphere of influence e.g., for suburban/commuting zones.

## References

[B1] Agyeman J. (2008). Toward a ‘just’ sustainability? Continuum 22, 751–756. 10.1080/10304310802452487

[B2] Agyeman J. Bullard R.D. Evans B. (2002). Exploring the nexus: bringing together sustainability, environmental justice and equity. Space Polity 6, 77–90. 10.1080/13562570220137907

[B3] Anguelovski I. Brand A. L. Connolly J. J. Corbera E. Kotsila P. Steil J.. (2020). Expanding the boundaries of justice in urban greening scholarship: toward an emancipatory, antisubordination, intersectional, and relational approach. Ann. Am. Assoc. Geogr. 29, 1–27. 10.1080/24694452.2020.1740579

[B4] Beck U. (1986). Risikogesellschaft. Frankfurt: Suhrkamp.

[B5] Butler P. (2020). Poor Housing Linked to High Covid-19 Death Rate in London Borough. Available online at: https://www.theguardian.com/world/2020/aug/17/poor-housing-linked-high-covid-19-death-rate-london-borough-brent

[B6] Chair A. (2020). Homes, Health, and COVID-19: How Poor Housing Adds to the Hardship of the Coronavirus Crisis. Available online at: https://www.smf.co.uk/commentary_podcasts/homes-health-and-covid-19-how-poor-housing-adds-to-the-hardship-of-the-coronavirus-crisis/

[B7] Clifford B. (2020). Available online at: https://theconversation.com/coronavirus-pandemic-puts-the-spotlight-on-poor-housing-quality-in-england-136453 (accessed July 14, 2020).

[B8] Crenshaw K. (2000). Background paper for the expert meeting on the gender-related aspects of race discrimination. Rev. Estud. Fem. 10:171. 10.1590/S0104-026X2002000100011

[B9] DGB (2020). Corona-Krise Verstärkt Soziale Ungleichheit. Available online at: https://www.dgb.de/themen/++co++6bf77ed6-9f34-11ea-9db2-525400e5a74a (accessed July 14, 2020).

[B10] Dick E. (2020). Available online at: https://www.die-gdi.de/en/the-current-column/article/how-the-corona-crisis-is-calling-into-question-the-right-to-the-city/ (accessed July 14, 2020).

[B11] Fainstein S. (2010). The Just City. Ithaca, NY: Cornell University Press.

[B12] Fisher M. Bubola E. (2020). Available online at: https://www.nytimes.com/2020/03/15/world/europe/coronavirus-inequality.html (accessed July 14, 2020).

[B13] Friedmann J. Bartsch M. (2020). Available online at: https://www.spiegel.de/panorama/gesellschaft/corona-risikofaktor-armut-das-sozialvirus-a-28a9400c-971e-453d-a775-b5cdad4aeb84 (accessed July 14, 2020).

[B14] Global Platform for the Right to the City (2020). Available online at: https://www.right2city.org/the-right-to-the-city-facing-covid-19/ (accessed July 14, 2020).

[B15] Haase D. Kabisch S. Haase A. Andersson E. Banzhaf E. Baró F.. (2017). Greening cities - To be socially inclusive? About the alleged paradox of society and ecology in cities. Habit. Int. 64, 41–48. 10.1016/j.habitatint.2017.04.005

[B16] Jones S. (2020). Available online at: https://www.theguardian.com/world/2020/sep/18/madrid-poor-spanish-capital-covid-19 (accessed September 21, 2020).

[B17] Kotsila P. (2020). Just and sustainable cities in the context of a changing climate, in Paper Held at the Webinar “Covid-19, Justice and Sustainability in Cities on 5 June 2020. Available online at: http://www.bcnuej.org/event/urbana-open-webinar-covid-19-pandemic/ (accessed July 15, 2020).

[B18] Kremer P. Haase A. Haase D. (2020). The future of urban sustainability: Smart, efficient, green or just? Introduction to the special issue. Sustain. Cities Soc. 51:101761. 10.1016/j.scs.2019.101761

[B19] Kronenberg J. Haase A. Łaszkiewicz E. Antal A. Baravikova A. Biernacka M.. (2020). Environmental justice in the context of urban green space availability, accessibility, and attractiveness in postsocialist cities. Cities 106:102862. 10.1016/j.cities.2020.102862

[B20] Langemeyer J. Connolly J. (2020). Weaving notions of justice into urban ecosystem services research and practice. Environ. Sci. Policy 109, 1–14. 10.1016/j.envsci.2020.03.021

[B21] Lutz H. (2014). “Intersectionality's (brilliant) career – How to understand the attraction of the concept,” in Working Paper Series ‘Gender, Diversity and Migration’ (Frankfurt: Goethe University).

[B22] Maalsen S. Martin C. Rogers D. Power E. (2020). Available online at: https://theconversation.com/why-housing-evictions-must-be-suspended-to-defend-us-against-coronavirus-1341480 (accessed July 14, 2020).

[B23] Malin S. Ryder S.S. (2018). Developing deeply intersectional justice scholarship. Environ. Sociol. 4, 1–7. 10.1080/23251042.2018.1446711

[B24] Menton M. Larrea C. Latorre S. Martinez-Alier J. Peck M. Temper L.. (2020). Environmental justice and the SDGs: from synergies to gaps and contradictions. Sustain. Sci. 1–6. 10.1007/s11625-020-00789-8

[B25] Mukherjee J. (2020). Wissenschaft: Lernt planetares Denken. DIE ZEIT No. 23/2020.

[B26] Nassehi A. Yüksel Y. (2020). Podcast “Geht es den Deutschen zu gut?” (Are the Germans doing too well?). Spiegel Online. Available online at: https://www.spiegel.de/politik/deutschland/corona-regeln-warum-sind-die-proteste-gerade-in-deutschland-so-heftig-a-749e924e-6d77-46ab-bb18-512ff0f7d57b (accessed September 22, 2020).

[B27] Samuelsson K. Barthel S. Colding J. Macassa G. Giusti M. (2020). Urban nature as a source of resilience during social distancing amidst the coronavirus pandemic. Landsc. Urban Plan. [Preprint]. 10.31219/osf.io/3wx5a

[B28] Sassen S. (2017). The city – a collective good? Brown J. World Aff. xxiii, 119–126.

[B29] Sauerbrey A. (2020). Available online at: https://www.tagesspiegel.de/politik/das-coronavirus-und-die-gesellschaft-keiner-hortet-sushi/25669244.html (accessed July 14, 2020).

[B30] Scheiwe H. (2000). Available online at: https://www.rnd.de/panorama/wohnbedingungen-der-tonnies-arbeiter-es-kann-nicht-sein-dass-menschen-wie-sklaven-gehalten-werden-455X6ROVAJDXLBHFJJI23MW4VQ.html (accessed July 14, 2020).

[B31] Schneidewind U. Baedeker C. Bierwirth A. Caplan A. Haake H. (2020). Näher-öffentlicher-agiler, in Eckpfeiler einer resilienten aezPost-Corona-Stadt, Working Paper. Available online at: https://wupperinst.org/fa/redaktion/downloads/publications/Post-Corona-Stadt.pdf (accessed June 9, 2020).

[B32] Simon D. (2020). Available online at: https://theconversation.com/cities-are-at-centre-of-coronavirus-pandemic-understanding-this-can-help-build-a-sustainable-equal-future-136440 (accessed July 14, 2020).

[B33] Teubner W. (2020). What is the New Normal? Challenges and Opportunities Beyond COVID-19. ICLEI News. Available online at: https://iclei-europe.org/news/?c=search&uid=LKhxRrBB (accessed July 14, 2020).

[B34] TNOC Blog Roundtable on Covid-19 (2020). Available online at: https://www.thenatureofcities.com/2020/05/03/covid-has-upended-all-the-normal-routines-in-our-lives-and-work-how-do-you-imagine-you-might-be-changed-by-it-both-professionally-but-also-personally-as-you-negotiate-a-new-post-virus-norm/ (accessed May 4, 2020).

[B35] Wennberg J. (2000). Available online at: https://www.aftonbladet.se/ledare/a/9vAO0M/virusdoden-far-inte-bli-en-klassfraga (accessed July 14, 2020).

[B36] Williams J.P. (2020). Available online at: https://www.usnews.com/news/healthiest-communities/articles/2020-03-19/coronavirus-could-crush-the-poor-and-homeless-advocates-warn (accessed July 14, 2020).

